# Spinal Orthoses Prescription for Vertebral Fragility Fractures by Italian Physical and Rehabilitation Medicine Physicians: The SPIN-VER Survey

**DOI:** 10.3390/healthcare9070892

**Published:** 2021-07-15

**Authors:** Alessandro de Sire, Antonio Ammendolia, Alessandra Gimigliano, Roberto Tiberi, Carlo Cisari, Marco Invernizzi

**Affiliations:** 1 Department of Medical and Surgical Sciences, University of Catanzaro “Magna Graecia”, 88100 Catanzaro, Italy; ammendolia@unicz.it; 2 Medical Direction, ASST Fatebenefratelli-Sacco, 20157 Milan, Italy; alessandra.gimigliano@gmail.com; 3 MyEvent SRL, Events Organization, Monte Porzio Catone, 00078 Rome, Italy; tiberi.r@myeventsrl.it; 4 Physical and Rehabilitative Medicine, Department of Health Sciences, University of Eastern Piedmont, 28100 Novara, Italy; cisari50@gmail.com (C.C.); marco.invernizzi@med.uniupo.it (M.I.); 5 Translational Medicine, Dipartimento Attività Integrate Ricerca e Innovazione (DAIRI), Azienda Ospedaliera SS, Antonio e Biagio e Cesare Arrigo, 15121 Alessandria, Italy

**Keywords:** vertebral fractures, spinal orthoses, osteoporosis, spine, disability, rehabilitation

## Abstract

Vertebral fragility fractures (VFFs) are the most common type of osteoporotic fractures, related to pain and disability. In this scenario, physical and rehabilitative medicine (PRM) physicians prescribe a patient-tailored rehabilitation plan, including spinal orthoses. However, there is a high heterogeneity in the clinical indications of spinal orthoses. Thus, the aim of this survey was to investigate common clinical practice in terms of the prescription of spinal orthoses. This nationwide cross-sectional survey recruited Italian PRM physicians commonly involved in the management of patients with VFFs. One hundred twenty-six PRM physicians completed the survey. The results showed that most PRM physicians prescribe spinal orthoses in outpatients suffering from VFFs (n = 106; 83.9%). The most prescribed spinal orthosis for acute VFF patients was the three-point rigid orthosis (n = 64; 50.8%), followed by the semirigid thoraco-lumbar orthosis (n: 20; 15.9%). However, most PRM physicians prescribed dynamic orthoses in outpatients with chronic VFFs (n = 66; 52.4%). Albeit that a correct management of VFFs is mandatory to improve pain and reduce disability, our findings highlighted uncertainty in the type of spinal orthosis prescription in both the acute and chronic VFF phase. Therefore, high-quality research trials are warranted to provide clear recommendations for the correct clinical management of VFF.

## 1. Introduction

The prevalence of aging-related diseases is a growing issue worldwide due to the increased average life expectancy, resulting in critical needs in socio-economic and health care systems [1]. In particular, the aging process might involve both bone and muscle tissues, with a resulting low bone mineral density (osteoporosis) and low muscle mass and function (sarcopenia) [2,3]. More in detail, osteoporosis is one of the most common age-related bone diseases that should be detected early, considering that a fragility fracture is considered as one of the most disabling complications in the elderly [4,5,6,7]. In this scenario, vertebral fragility fractures (VFFs) are defined as fractures occurring without a trauma or after a fall from a standing position [8]. They are the most common type of osteoporotic fractures, occurring in about 30–50% of osteoporotic patients [9,10]. It must be noted that a VFF could be considered as a major risk for subsequent VFFs, as shown by the increased risk of a second VFF by four to seven times after the first VFF [11,12]. The main clinical manifestation might be represented by back pain, with consequent impairment in physical function, disability, and restricted independence in activities of daily living (ADL) [5,6,9,11,12,13,14]. Lumbar spine and the dorsal-lumbar passage are considered as the most frequent VFF localization [15]. To date, pharmacological therapy is considered as the main intervention for patients with VFFs, considering the strong evidence on available drugs targeting bone metabolism [15]. However, the current management is still evolving as pharmacological new therapeutic strategies consist of molecules that enhance bone formation or inhibit bone resorption [16,17,18]. Moreover, several issues might interfere with the effectiveness of anti-osteoporotic drugs, such as poor adherence to therapy [19]. In this scenario, physical and rehabilitative medicine (PRM) physicians commonly prescribe a patient-tailored rehabilitation plan (i.e., early mobilization exercises, strengthening and balance training) in cooperation with physiotherapists to improve functioning and reduce the risk of falling in patients with VFFs [4,20]. Specifically designed therapeutic exercises with rapid and repetitive movements that load bones from many directions might be beneficial for these subjects, preventing also new VFFs [21]. Furthermore, another non-pharmacological therapeutic option, commonly prescribed by PRM physicians for patients with VFFs are the spinal orthoses [22]. They are used especially in acute or subacute phases to reduce excessive trunk flexion, to obtain an adequate posture, to improve pulmonary function and to reduce back pain [23,24,25]. Several models have been proposed based on mechanical properties (rigid, semi-rigid, or dynamic), and different studies have proposed spinal orthoses as an effective therapeutic intervention on pain relief for acute VFFs management [23]. This could be achieved by limiting spinal flexion and the load of forces applied on the anterior column and vertebral body [26]. Moreover, spinal orthoses might induce an upward shift of the center of mass of the trunk in the sagittal plane, normally induced by muscles and ligaments but frequently impaired in patients with VFFs [27]. In 2011, Pfeifer at al. [25] showed the effectiveness of a dynamic spinal orthosis, consisting of a belt and Velcro system, with traction elements around the pelvis and shoulders, with or without a posterior flexible bar, in reducing back pain and improving muscle strength of the trunk and abdominal extensors in VFF patients. Other studies have confirmed the potential positive effects on the muscle strength of the trunk extensors [27,28], as well as the better compliance with respect to rigid and semi-rigid orthoses [29]; however, long-term use of spinal orthoses is not recommended due to the risk of trunk extensor atrophy [30,31,32]. To date, there is still little evidence about the effectiveness of spinal orthoses in the management of VFFs in terms of pain reduction, improvement of independence in ADL and health-related quality of life (HRQoL) [23,24,25]. Moreover, the overall low quality of the studies available and their controversial findings do not allow for making any strong conclusions [23,33]. Lastly, there is high heterogeneity in the clinical indications of spinal orthoses, with a large variability of treatment protocols and outcome measures assessed [28,34,35]. As a result, the appropriate prescription of the type, timing and duration of spinal orthoses is frequently based on the clinical experience of the PRM physicians rather than supported by evidence-based medicine.

In this complex scenario, a previous survey was performed by Caitriona et al. [22] on spinal orthoses prescription in VFF patients. However, the authors assessed VFFs in hospital settings, focusing on a large variability of therapeutic interventions, without a precise assessment of spinal orthoses type, time, and dismission after VFFs.

To date, to the best of our knowledge, no previous surveys have assessed spinal orthoses prescription by PRM specialists in the outpatient settings for patients with VFFs.

In light of these considerations, the aim of this nationwide cross-sectional survey was to characterize the common practice in terms of prescription of spinal orthoses in outpatients affected by VFFs to provide useful information starting from the experience of an Italian cohort of PRM physicians.

## 2. Materials and Methods

### 2.1. Participants

In this nationwide cross-sectional survey, entitled “Spinal orthoses Prescription IN VERtebral fractures (SPIN-VER)” Survey, we involved a cohort of Italian PRM physicians commonly involved in the management of patients with VFFs in their outpatient clinics. All study participants were recruited from April 2020 to May 2020 through an email recruitment strategy (starting from recent National Congresses on this topic) and were asked to participate in an online questionnaire, after a detailed description of the survey and a previous authorization by the physicians to be contacted for survey purposes.

Inclusion criteria were: (a) physicians specializing in PRM; (b) physicians working in outpatient clinics; (c) physicians involved in the management of patients with VFFs; (d) consent to share their data with the researchers (that guaranteed privacy protection and permission for distribution of the survey). The online questionnaire registration aimed at gathering sociodemographic and professional information including age, gender, working region, work experience, and years of activity. This study was approved by the Institutional Review Board (61/10 P.392) and was performed in accordance with pertinent National regulatory requirements. All participants were asked to carefully read and sign an informed consent form before collecting the data and privacy protection was guaranteed by the study investigators.

### 2.2. Survey

All participants were asked to complete a 13-item questionnaire built for the SPIN-VER Survey with the aim to investigate the clinical management of patients affected by VFFs referring to the outpatient clinics.

The survey questionnaire was developed by a technical expert panel consisting of four PRM professors with expertise in osteoporosis and fragility fractures management and two medical experts in data organization and analysis. The present study was conducted in accordance with previous well-conducted cross-sectional surveys present in the scientific literature [36,37,38,39].

The questionnaire consists of the following domains: (A) information on outpatients assessed by the physicians in their common clinical practice (questions 1–5); (B) information on spinal orthoses prescription by the physicians in patients with back pain without VFFs (questions 6–7); (C) information on spinal orthoses prescription by the physicians in patients with acute VFFs (questions 8–10); (D) information on spinal orthoses prescription by the physicians in patients with chronic VFFs (questions 11–12); (E) information on pharmacological therapy (i.e., bisphosphonates, denosumab, teriparatide), calcium and vitamin D supplementation, therapeutic exercise, and instrumental physical therapies prescription by the physicians in patients with VFFs (question 13). The last question is the only one that allowed more than one answer. The questionnaire is described in detail in Table 1.

### 2.3. Statistical Analysis

All data were recorded, categorized, and subsequently analyzed by Graphpad Prism 7.0 (GraphPad Software, Inc., San Diego, CA, USA). Descriptive statistics have been used to analyze the data. Results are presented as mean and standard deviation for continuous variables, and counts and percentages for dichotomous, nominal, and ordinal variables. The distribution of numerical data was summarized through figures and tables. The correlation between the participant’s characteristics and the survey responses was investigated by Spearman’s correlations. A *p*-value less than 0.05 was considered statistically significant.

## 3. Results

We included data collected from 126 PRM physicians (mean age: 44.2 ± 13.6 years old; female/male: 49/77) that completed the survey. Most participants reported fewer than 5 years’ experience, 54 (42.9%) as PRM specialists, while 29 specialists (18.2%) reported more than 20 years’ experience. Most respondents (n: 83; 65.9%) were working in hospital centers as staff physicians, while the other physicians worked in a private practice (n: 22; 17.4%). Twenty-one participants (16.7%) did not indicate their professional role. The study sample came from all over Italy: 54 (42.9%) were from the Northern Italy, 23 (18.2%) from Central Italy, whereas 49 (38.9%) were from Southern Italy. Almost half of the physicians involved in the survey reported to assess more than 100 patients per month with 14.3% of participants managing over 200 patients per month. Seventy-one participants (56.3%) reported a prevalence of 20–50% of osteoporotic patients and 13 (10.3%) reported a diagnosis of osteoporosis in even more than 50% of their patients. The 66.7% of participants reported the absence of VFFs (at X-ray examination) in low back pain patients in more than 20% of cases. All the above-mentioned information on outpatients assessed by the physicians in their common clinical practice, referring to the “domain A” of the survey, were reported in detail by Table 2.

**Figure 1:**
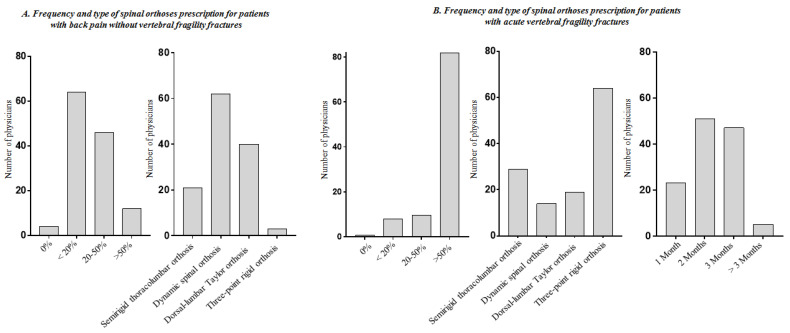
Information on spinal orthoses prescription by the physicians in patients with back pain without vertebral fragility fractures and with acute vertebral fragility fractures (questions included in domains B and C of the survey).

Sixty-four PRM physicians (50.8%) reported to prescribe spinal orthoses in less than 20% of cases of patients affected by back pain without VFFs, with a high variability in terms of orthosis type. Dynamic spinal orthosis was the most frequently prescribed by PRM physicians (62 cases, 49.2%). Figure 1A described further details. On the other hand, a high rate of spinal orthoses prescription was found in patients affected by VFFs, and 83.9% of PRM specialists prescribed spinal orthoses in more than 50% of VFFs patients. The most prescribed spinal orthosis in the common clinical practice was the three-point rigid orthosis (n = 64; 50.8%), followed by the semi-rigid thoraco-lumbar orthosis (n = 20; 15.9%). A discrete heterogeneity has been found concerning the timing for discontinuance of wearing the orthosis in patients with acute VFFs. Twenty-three PRM physicians (18.2%) recommended discontinuance of the spinal orthosis after 1 month, 51 (40.5%) after 2 months, 47 (37.3%) after 3 months, and 5 (4.0%) after more than 3 months (see Figure 1B for further details).

**Figure 2:**
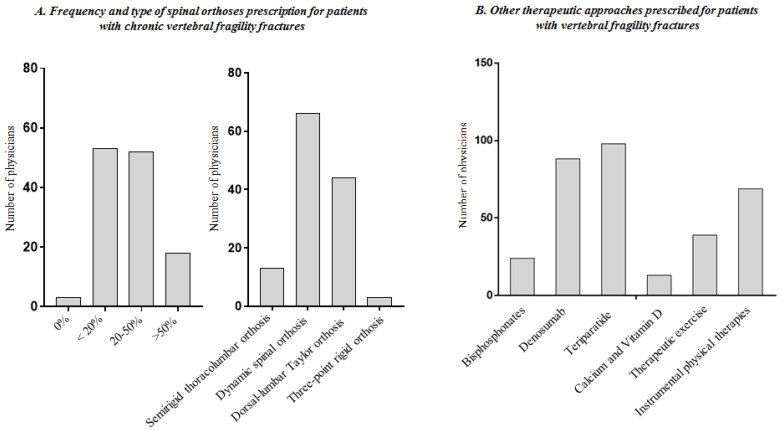
Information on prescription by the physicians in patients with back pain without vertebral fragility fractures and with acute vertebral fragility fractures (questions included in the domains D and E of the survey).

Moreover, we investigated if PRM physicians prescribed spinal orthoses even in patients affected by chronic VFFs. Most physicians (n = 108; 85.7%) affirmed to prescribe spinal orthoses in less than 50% of cases. Dynamic spinal orthoses resulted to be the most frequently prescribed (n = 66; 52.4%) followed by dorsal-lumbar Taylor spinal orthoses (n = 44; 34.9%) (see Figure 2A for further details). In relation to the other therapeutic approaches prescribed by PRM physicians in patients with VFFs, calcium and vitamin D supplementation were the most common intervention (n: 113; 89.6%), while the most common pharmacological therapy prescribed was represented by bisphosphonates (n: 102; 80.9%), followed by denosumab (n: 38; 30.2%), and teriparatide (n: 28; 22.2%). Notably, therapeutic exercise was prescribed by 87 PRM physicians (69.0%) in the common clinical practice; lastly, instrumental physical therapies (e.g., ultrasounds, laser therapy, transcutaneous electrical nerve stimulation, etc.) were prescribed by only 57 PRM physicians (45.2%) (see Figure 2B for further details).

Lastly, we performed a correlation analysis which showed a significant correlation between the age of PRM specialists involved in this survey and the prescription of spinal orthoses in patients suffering from LBP without VFFs (rho: 0.207; *p* < 0.01) and patients affected by chronic VFFs (rho: 0.446; *p* < 0.001). However, no significant correlation was found between the age of PRM specialists and the spinal orthoses prescription after acute VFFs (rho: 0.046; *p* > 0.05), even though there was a higher trend in older physicians.

## 4. Discussion

The present nationwide cross-sectional survey aimed to assess the clinical management of patients with VFFs, highlighting key clinical decision-making challenges in spinal orthoses prescription by PRM physicians, despite the large gap of knowledge concerning spinal orthoses effectiveness in terms of pain reduction, functioning improvement, and HRQoL improvement. Considering the wide presence of PRM outpatients affected by back pain [13], our findings showed that spinal orthoses were prescribed by 83.9% of PRM physicians in more than half of patients with acute VFFs, while the most prescribed was the three-point rigid orthosis (*n* = 64; 50.8%). It is widely accepted in the common clinical practice that one of the primary goals in the complex management of patients with acute VFFs is pain relief, followed by postural control achieved by inhibiting the anterior spinal flexion. However, the current literature describes only one low-quality study supporting spinal bracing interventions for pain and posture improvement in VFF patients [23,40,41,42]. Nevertheless, some studies assessed long-term effects of brace wearing compared with early mobilization in patients with stable VFFs, showing similar results in terms of pain relief and disability reduction [43,44,45]. Pawardhan et al. reported that rigid thoracolumbar braces in acute VFFs could induce trunk muscles atrophy and restrict respiration, suggesting a harmful role of this treatment in these patients [46]. These controversial data might be also due to the poor tolerance of spinal orthoses as reported by older people, given the cumbersome intrinsic nature of braces [45,47]. However, our survey showed a positive trend of spinal orthosis prescription in current clinical practice and 50.8% of physicians prescribed three-point rigid orthoses in patients with acute VFFs. These findings evidenced some widely spread therapeutic pathways in the current clinical practice, albeit not fully supported by strong evidence, given the large gap in the currently available literature. However, a recent prospective comparative study, performed by Meccariello et al. [29] on 140 older VFFs patients showed that a dynamic spinal orthosis group had significantly better outcomes (*p* < 0.05) in terms of pain, disability, and respiratory function than a three-point spinal orthosis group at 3- and 6-month follow-ups. They suggested that biofeedback activation of back muscles might play a key role in improving functional outcome with dynamic orthosis. A recent meta-analysis performed by Jin et al. [41] showed that dynamic braces could be supported by a better quality of evidence in pain relief among different types of spinal orthoses. Another controversial topic debated in literature is the timing of brace wearing until discontinuance. In this context, the most common complications of prolonged spinal orthoses wearing might be spinal extensor activity reduction [30,31] and muscle atrophy [32]. Kim et al. reported that spinal orthoses might have positive effects in pain relief in the first 6–8 weeks [48]. On the other hand, several studies reported side effects in patients wearing spinal orthoses for 3 months with controversial results [25,26,29,34]. The large majority of PRM physicians (80.3%) involved in our nationwide cross-sectional survey suggested to discontinue the wearing of spinal orthoses after 2 or 3 months from VFFs onset. Few studies have evaluated the effects of spinal orthoses in patients with chronic VFFs, showing that dynamic spinal orthosis seemed to provide promising results in terms of pain relief and functioning improvement [26,28]. Moreover, the so-called “biofeedback” has been proposed as the mechanism of action underpinning these results, hypothesizing that spinal orthoses might actively stimulate the contraction of back extensor muscles through a lower degree of immobilization [26,29]. Although only 20% of PRM physicians participating in our study affirmed to prescribe spinal orthoses in more than 50% of their patients with chronic VFFs, dynamic spinal orthoses resulted to be the most frequently prescribed (52.4%), in line with the current available literature [26,28].

Taken together, the data reported underlined controversial findings reported in the current literature [25,26,29,34]. Therefore, our findings might improve knowledge in clinical practice of PRM specialists paving the way to future research on this topic. Improving the standardization of the therapeutic approach represents a critical issue in the studies assessing the effects of spinal orthosis in VFFs. In addition, the progress of VFFs should be strictly monitored given the lack of data assessing the optimal time of bracing dismission and the high risk of VFFs recurrence or further onset of VFFs in patients with osteoporosis.

In the complex scenario of the management of osteoporotic patients, the PRM physicians involved demonstrated the provision of appropriate pharmacological anti-osteoporosis treatment with a higher prescription of bisphosphonates (80.9%) as the first-line approach for patients with less than three VFFs. Moreover, it should be noted how calcium, vitamin D, and amino acids supplementation should be considered in a multidisciplinary integrated intervention combined with lifestyle education to prevent or to counteract the developing of osteosarcopenia [3,49,50,51,52]. Almost 70% of the PRM physicians involved prescribed therapeutic exercise in their clinical practice; these findings are in line with the available literature suggesting its beneficial effects of exercise in terms of pain, posture, physical performance, and HRQoL [53,54,55,56,57]. In this scenario, a recent Cochrane Systematic Review performed by Gibbs et al. [58] supported exercise therapy as an effective intervention in functional outcomes improvement in patients suffering from osteoporotic vertebral fractures with moderate evidence. Several countries need PRM physicians referring their patients with VFFs to physiotherapists for an adequate rehabilitative treatment.

To our knowledge, this is the first study designed to assess the effects of spinal orthoses in a multitarget rehabilitation approach including pain management, anti-osteoporotic drugs, and physical exercise. Therefore, further studies are warranted to understand the potential synergic approach of comprehensive therapy in patients suffering from VFFs in terms of functional outcomes and HRQoL.

### Study Limitations

We are aware that this study is not free from limitations. First, answers given by the PRM specialists provided low-quality evidence (expert opinion) according to the Oxford CEBM Levels of Evidence [59]. This expert opinion was recorded by a sample of voluntary PRM physicians that might result in a selection bias. Participants might have been concerned about the anonymity of their answers with a negative implication in terms of response rate. Moreover, other physicians might be involved in the management of patients with VFFs (i.e., orthopedics, rheumatologists, geriatrics, neurosurgeons, etc.), and their clinical experience was not recorded. However, it should be noted that the present study showed for the first time in literature the heterogeneity in the clinical management of spinal orthosis prescription in the PRM setting, highlighting the limitation in current evidence, and emphasizing the needing for further studies.

However, given the large sample and the large gap of knowledge in this field, our findings might support clinicians to perform a standardized intervention in patients with VFFs, considering the complications that might result from this highly disabling condition.

## 5. Conclusions

A correct management of VFFs is mandatory to improve pain and reduce disability in fragile osteoporotic patients. The results of the SPIN-VER survey showed that most of the PRM physicians prescribed spinal orthoses in outpatients suffering from VFFs, even though this therapeutic intervention is not fully supported by evidence, partly due to the large gap in the current literature. However, we highlighted a substantial uncertainty in the type of spinal orthosis prescription (in both the acute and chronic VFF phase) and in the timing for dismission. The most prescribed spinal orthosis for acute VFF patients was the three-point rigid orthosis, although only dynamic orthoses had a real scientific and evidence-based basis for these patients. However, most PRM physicians prescribed dynamic orthoses in outpatients with chronic VFFs, in accordance with the available scientific evidence. In conclusion, the large heterogeneity of clinical presentations of patients with VFFs represents important barriers in the adequate management of these subjects. The present study underlined for the first time in literature the heterogeneity of spinal orthoses prescription by PRM specialists in VFF patients. This was mainly due to the lack of specific indications, emphasizing the need for high-quality studies investigating the role of dynamic spinal orthoses in VFFs patients to provide clear recommendations for the correct clinical management of this detrimental condition.

## Figures and Tables

**Table 1:** A 13-item questionnaire aimed to investigate the clinical management of patients affected by vertebral fragility fractures referring to physiatric outpatient clinics.

*Domain A*
1. How many patients do you visit in your outpatient monthly?
2. What percentage of patients with osteoporosis do you visit in your outpatient?
3. What percentage of patients with back pain do you visit in your outpatient?
4. What percentage of patients with acute vertebral fragility fractures do you visit in your outpatient?
5. What percentage of patients with chronic vertebral fragility fractures do you visit in your outpatient?
*Domain B*
6. What percentage of patients with back pain without vertebral fragility fractures do you prescribe with spinal orthoses?
7. Which is the spinal orthosis most prescribed by you in the above-mentioned patients?
*Domain C*
8. What percentage of patients with acute vertebral fragility fractures do you prescribe with spinal orthoses?
9. Which is the spinal orthosis most prescribed by you in the above-mentioned patients?
10. What is the timing of spinal orthosis dismission that you suggest in the above-mentioned patients?
*Domain D*
11. What percentage of patients with chronic vertebral fragility fractures do you prescribe with spinal orthoses?
12. What is the spinal orthosis most prescribed in these patients?
*Domain E*
13. Which treatment do you mostly prescribe in patients with vertebral fragility fractures?

The questionnaire consists of 5 domains: (A) information on outpatients assessed by the physicians in their common clinical practice; (B) information on spinal orthoses prescription by the physicians in patients with back pain without vertebral fragility fractures; (C) information on spinal orthoses prescription by the physicians in patients with acute vertebral fragility fractures; (D) information on spinal orthoses prescription by the physicians in patients with chronic vertebral fragility fractures; (E) information on pharmacological therapy, calcium and vitamin D supplementation, therapeutic exercise, and instrumental physical therapies prescription by the physicians in patients with vertebral fragility fractures.

**Table 2:** Information on outpatients assessed by the physicians in their common clinical practice (questions included in the domain A of the survey).

	PRM Physicians (n = 126)
Number of outpatients assessed per month	
<50	32 (25.4)
50–100	35 (27.8)
100–200	41 (32.5)
>200	18 (14.3)
Patients with diagnosis of osteoporosis	
0%	1 (0.8)
<20%	41 (32.5)
20–50%	71 (56.3)
>50%	13 (10.3)
Patients with back pain without VFFs	
0%	0 (0.0)
<20%	23 (18.3)
20–50%	64 (50.8)
>50%	39 (30.9)
Patients with acute VFFs	
0%	8 (6.3)
<20%	105 (83.3)
20–50%	12 (9.5)
>50%	1 (0.8)
Patients with chronic VFFs	
0%	0 (0.0)
<20%	49 (38.9)
20–50%	61 (48.4)
>50%	16 (12.7)

The variables are expressed as counts (percentages). Abbreviations: PRM: Physical and Rehabilitative Medicine. VFFs: Vertebral Fragility Fractures.

## Data Availability

Dataset is available on request.
